# The Modern Jew

**Published:** 1918-01-05

**Authors:** 


### THE MODERN JEW.

### A Sociological and Biological Study.

In ordinary conversation one of the first rules to be followed is to agree instantly and not to go deeply into the subject under discussion. So that if an interesting drawing-room or club talker were to string together five names of great contemporary (most of them of probably more than contemporary) distinction, he would enjoy quite a conversational triumph when going on to argue that the nation to which all five belonged was now, with the removal of old-time disabilities, proving its innate intellectual superiority to European races. To allow this king of a somewhat submissive company his due, his choices might give anyone a moment's pause, seeing that they would probably be—Ehrlich, Metchnikoff, Bergson, Freud, and Kerensky. It might sound like carping to object that genius, which the first two certainly had, is a sport, essentially unrepresentative of the stock; or that if you must not indict a nation then equally you cannot apotheosise one. It would hardly bring conviction, either, to deny that Parisian philosophy could ever fail of its resemblance to Brummagem silver, that Freud's international fame is other than a bubble blown from dirty water, and Kerensky other than a transient and embarrassed phantom. Rejoinders of this sort would seem ill-mannered contrariety to the ladies, or the sitters in big chairs, already spoken of. They might not wholly settle the matter even with knowledgeable people.' At all events (these might say) one will find interest in a good account of Jewish tradition as bearing upon the present and future position of that race. This is how we have felt ourselves, to the point of giving up, in these pinching times, some considerable amount of space to a book * by a medical man which probably aspires to serve the purpose stated, leaving it to be understood that the child is father to the man. Or more likely the author's nationality and pediatrical experience together suggested the subject to him. However, it is unfortunate that the particular reference is not marked enough: perhaps it was not possible to bring out anything much new or significant with regard to Jewish childhood.

Skipping expositions, well enough done, and perhaps necessary to a plea for the insight of early Hebrew savants, but damaging to the tone of the work, of things like Mendelism, mathematical problems, and biometry, we note that the Talmud says that fame and greatness are not meant for women. On page 2 the woman comes into her own again. More important than this, what a lesson here for the modern luxurious childless husband and wife!

> Then what could death do, if thou should'st depart
> Leaving thee living in posterity?

says Shakespeare. Well, sages have always preached and ordinary people gone their own way notwithstanding. But the former Jew did on the whole fulfil his duty to the race by leaving progeny. The present one, contrary to what is widely believed, does not: Jewish marriage and birth rates are *lower* than those of Gentiles. These errors it is useful to get right about. In mixed marriages the Jewish element is *not* (as many, by the way, say too gipsy blood is) prepotent. But Jews do live longer, and their children have a much less infantile mortality, though probably more because of parental care than from any innate high physical vitality. Also they, partly for these same reasons of health, attend school more regularly. Like the Scotch, the Jews are strong for education, at least (a wise provision Mr. Fisher should note) after six years of age. But distinction such as that attained by the great French, Russian, and German Jews cited at the beginning of these remarks is independent of education or of clannishness coupled with a strong resolve to get on in the world. It will be interesting to see if the phenomenon of preeminent Jews continues. *Item.*—Jews don't object to vaccination: it is a trifle to circumcision; and perhaps education tells again, too. *Item.*—Psycho-analysts would be glad of the Rabbinical maxim—"More than the calf wishes to suck does the cow wish to suckle." *Item.*—This is a striking saying—"Man comes into the world with closed hands, as though claiming ownership of everything; but he leaves it with hands open and limp, as if to show he takes nothing with him."

The book is a maze but not without a plan; and not without, either, plain signs of ability both literary and scientific. Though a maze, it did not need the introduction. That nearly all "forewords" are a mistake, all parties concerned, except perhaps publishers, should have reason to know by now.

* *The Jewish Child: Its History, Folklore, Biology, and Sociology.* By W. M. Feldman, M.B., B.S. Lond. With an Introduction by Sir Jas. Crichton Browne. (London: Baillière, Tindall, and Cox. 1917.)

